# The future of pediatric dentistry education and curricula: a Chilean perspective

**DOI:** 10.1186/s12903-016-0251-7

**Published:** 2016-07-18

**Authors:** Rodrigo Mariño, Francisco Ramos-Gómez, David John Manton, Juan Eduardo Onetto, Fernando Hugo, Carlos Alberto Feldens, Raman Bedi, Sergio Uribe, Gisela Zillmann

**Affiliations:** Melbourne Dental School, University of Melbourne, Melbourne, Australia; School of Dentistry, University of California-Los Angeles, Los Angeles, CA USA; Facultad de Odontología, Universidad de Valparaiso, Valparaiso, Chile; School of Dentistry, Universidade Federal do Rio Grande do Sul, Porto Alegre, Brazil; School of Dentistry, Universidade Luterana do Brasil, Canoas, RS Brazil; Dental Institute, Kings College London, London, UK; Dental School, Universidad Austral de Chile, Valdivia, Chile; Facultad de Odontología, Universidad de Chile, Santiago, Chile; Oral Health Cooperative Research Centre, University of Melbourne, Melbourne, Australia

**Keywords:** Paediatric dental education, Chile, Curriculum

## Abstract

**Background:**

A meeting was organised to consolidate a network of researchers and academics from Australia, Brazil, Chile, the UK and the USA, relating to Early Childhood Caries (ECC) and Dental Trauma (DT). As part of this meeting, a dedicated session was held on the future of paediatric dental education and curricula. Twenty-four paediatric dentistry (PD) academics, representing eight Chilean dental schools, and three international specialists (from Brazil and Latvia) participated in group discussions facilitated by five members of the ECC/DT International Collaborative Network. Data were collected from group discussions which followed themes developed as guides to identify key issues associated with paediatric dentistry education, training and research.

**Discussion:**

Participants discussed current PD dental curricula in Chile, experiences in educating new cohorts of oral health care providers, and the outcomes of existing efforts in education and research in PD. They also, identified challenges, opportunities and areas in need of further development.

**Summary:**

This paper provides an introspective analysis of the education and training of PD in Chile; describes the input provided by participants into pediatric dentistry education and curricula; and sets out some key priorities for action with suggested directions to best prepare the future dental workforce to maximise oral health outcomes for children. Immediate priorities for action in paediatric dentistry in Chile were proposed.

## Background

In many countries over the last 25 years, there has been a significant decline in dental caries experience, as well as in other oral health problems among children [1–4]. Despite these improvements in the oral health of the population, numerous challenges in reducing inequalities in oral health status remain [5, 6]. Notably, certain groups in the community continue to be at a higher risk of dental caries [7]. Oral health conditions associated with poverty, family structure and other social and psychosocial factors are appearing with increased frequency [4, 6]. Thus, despite general improvement in child oral health in several countries [8, 9], early childhood caries (ECC) still represents a serious public health problem and a challenge for the dental profession. The treatment of ECC is expensive for both the individual and the society, as the disease carries with it a personal cost of morbidity and impacts on the ability to thrive [10]. ECC in its most severe form may require comprehensive management under general anaesthesia, which by itself does not cure or resolve the risk of further ECC [11, 12] More importantly, ECC is associated with a decreased quality of life in preschool children and their parents/caregivers [13, 14].

Additionally, in the last century, injury surpassed disease as the leading cause of childhood mortality and disability in developed as well as developing countries [15–17]. Dental trauma (DT) has been reported as making up 35 % of facial injuries. Between 31 and 39 % of dental emergencies are consequences of DT [18]. Consequently, DT is recognised as a major dental public health problem worldwide [19].

Thus, ECC and DT represent two common and serious public health problems. As such, they are important challenges to the oral health profession and the wider society. Furthermore, they raise questions about the current model of training general dental practitioners, as well as dentists specialising in paediatric dentistry (PD) and endodontics. Dental schools need to respond to these epidemiological challenges with innovative curricula and enhanced education methods. This supports the need to assess whether graduates have the expected skills, knowledge and competencies that dentists and PD specialists need to address the oral health needs of a diverse paediatric population.

The Faculty of Dentistry, University of Valparaiso, Chile, and The Melbourne Dental School, University of Melbourne, Australia organised a workshop to encourage collaboration among researchers and academics from Australia, Brazil, Chile, the UK, and the USA. The workshop took place in the Valparaiso, Chile in April 2014. As part of this workshop, a dedicated session on the future of paediatric dental education and curricula was organised with academics, oral health professionals and postgraduate students with interest in PD from Brazil, Chile, Latvia, the UK and the USA. The purpose of the session was to review and discuss the status, structure and content of current dental curricula in paediatric dentistry in Chile; describe Chilean standards in paediatric education; and limitations in preparing new cohorts of oral health care providers for practice. In the session existing efforts in education and research in this field were discussed, and areas of need for further development identified, with challenges and opportunities in these areas pinpointed from a Chilean perspective.

### Material and methods

A letter was sent to heads of paediatric dentistry in all Chilean dental schools/faculties inviting them to participate in a group discussion session about the future of pediatric education in Chile, or to nominate a member of his/her teaching team. The session was attended by 24 paediatric dentists, representing eight Chilean dental schools/faculties, and three international specialists (from Brazil, Latvia and Scotland), plus another six international workshop participants.

To accomplish the session’s goals, participants worked in five groups of 5–6 participants each with one group facilitator. A set of themes was developed during the organisational stage of the meeting as a guide to identify key issues around paediatric dentistry education, training and research. The facilitators began the discussions by reminding participants that the discussion material would be the subject of a publication and asked for verbal consent to include the comments anonymously. This was followed by a brief self-introduction by participants and a chance to describe his/her local experiences with undergraduate and postgraduate education in paediatric dentistry. After the introductions, each group discussed the various topics and themes presented in the agenda and made a report to the plenary session that followed. After discussion of these reports in the plenary session, issues emerged, which cut across all discussion groups, and reflected the sentiments of the full group. A debate ensued in the way participants discussed and decided on issues raised during the group discussions and the way to arrive to consensus on differences and reflect the sentiments of the whole group as a coherent document.

Data reported here summarises authors’ notes (RM, FRG, JO, FH, CF) from the group discussions about paediatric dental education in Chile. Therefore, they are purely observational, with no element of intervention. In addition, they do not reflect the result of medical or health research and, as such, they did not require approval or review from an ethics committee. Nonetheless, this publication follows the World Medical Association Declaration of Helsinki guidelines and is in accordance with Australian and Chilean practices and approvals for projects that do not involve access to or collection of private, sensitive or health data. Participants in the workshop were advised that the groups’ discussions would be the subject of a paper, but did not review or approve the final version of the manuscript.

## Results

Major topics and issues emerging in the discussions centred on three major interrelated categories. These include: i) the need to continuously, or periodically, review the status, structure and content of current undergraduate/graduate dental curricula in paediatric dentistry (PD) in Chile, and continuing professional development contents, in view of advancements in science and technology, new epidemiological data, national health policies and politics etc.; ii) how to prepare educators, scientists and researchers in PD; and iii) in order to move forward, identify challenges and opportunities relevant to these areas. Please see Table 1.

**Table 1:** Summary of main outcomes

• There is a need to review the undergraduate and graduate dental curricula in paediatric dentistry.
• Undergraduate training and education in paediatric dentistry is fragmented with wide variability, lack of uniformity as well as duplication of contents within the different oral disciplines in the curriculum contents offered by dental schools.
• Patient-oriented models should be developed, focusing on the health team in the context of the family and the community.
• Competencies regarding medically complex patients should be incorporated in postgraduate training.
• There is a need for evidence-based continuing professional development for general dental practitioners and specialist dentists.
• Research plays an important role in providing the scientific basis for paediatric dentistry teaching and practice.
• New opportunities include formal and informal mentoring provided by well-trained researchers as well as expansion in education technology to enhance lifelong learning.

## Paediatric dentistry training and education

The education process was conceptualised as a continuum of related experiences that begins during undergraduate education and builds throughout the professional life of a dentist, entailing a set of knowledge, skills, competences and attitudes towards PD to be covered as undergraduate (UG), graduate, postgraduate (PG), or as continuing professional development (CPD) courses.

### Undergraduate training and education: current challenges

Undergraduate PD is taught as clinical courses mainly in the fourth and fifth years of a 6-year degree course in Chile. Pre-clinical activities are commonly in the third year, and in the fourth year students start performing clinical procedures on children. Participants agreed that undergraduate PD programmes need to develop general dental practitioners (GDP) who are able to manage the oral health of pre-school children effectively, including prevention, early intervention and treatment of simple issues and more complex cases. It was noted that although some oral health problems (e.g. ECC and DT) might be complex and multifactorial in nature, their prevention and management should be emphasised as part of the UG curriculum.

However, according to participants in the current curriculum, training and education is fragmented, with wide variability and lack of uniformity in the curriculum contents offered by the different dental schools represented in this session. This discussion indicated that, although most dental schools gave special attention to some aspects of cariology, other relevant topics still needed to be addressed. Regarding DT, with the exception of one school that has undergraduate modules (University of Valparaiso, Chile), there was minimal teaching of DT.

Participants agreed that a need exists for greater emphasis on developing patient-oriented models, focussing on the health team to deliver better outcomes. It was also agreed that together with acquiring clinical competencies, dental students should be able to assess children in the context of the family and the community. In particular, participants highlighted the need to incorporate teaching about social determinants of health (i.e., gender, race/ethnic discrimination, etc.), about children’s rights and local laws and practices, and other complementary skills to provide a solid base for advocacy [20]. Advocacy was seen as a most powerful instrument to address social, economic and environmental inequalities. At the same time, social determinants of health and advocacy were perceived as difficult to consider appropriately within the context of an unbalanced biomedical approach.

There was also common agreement that avoiding the duplication of contents within the different oral disciplines would be of great advantage. Participants provided an overview of contents from existing undergraduate programs that could be substantially modified, while still achieving competency and high quality oral health outcomes. This included topics which were considered time consuming, and comprised a significant proportion of undergraduate education (e.g. in maxillofacial surgery, orthodontics, restorative dentistry, and complex oral rehabilitation).

### Postgraduate training and education: lack of consensus and accreditation

In regard to specialist training in PD, participants highlighted the increasing offering of courses, with great variability in terms of length, content, and course credits per unit. In most Chilean programs, specialist training takes 2 years, but there are also 1-year programs being offered. Furthermore, none of these programs are at the master’s level, but exist as professional specialization programs. Additionally, the lack of accreditation in Chile for postgraduate paediatric dentistry education programs was noted.

The lack of consensus and accreditation,^1^ and the need to ‘rethink’ the education in this specialty in Chile to incorporate other competencies, in particular, those needed to treat medically complex patients was discussed. Participants agreed that the number of children, adolescents and adults with special needs and chronic conditions has increased over the last few decades. In addition, for participants, it was not unusual that PD treat these patients. The need to expand PG education and to develop postgraduate programs in dental traumatology was also emphasized. One postgraduate program was identified which includes DT modules. The University of Valparaiso offers a postgraduate Diploma in dental trauma, as well as a diploma in cariology and preventive dentistry.

### Continuing professional development: standardization of contents

Since the body of health knowledge is constantly expanding, GDPs and specialist dentists need to maintain lifelong learning. The maintenance and further development of competence through continuing professional development were seen as essential for the provision of state-of-the-art, high quality oral health care. Dental practitioners need to confidently manage paediatric problems in the context of evidence-based practice and have prompt access to new developments and information. This would require equipping GDPs and specialists with the skills and abilities to search, select, critically analyse and apply valid and relevant evidence-based information to maintain lifelong learning.

Participants called for an expansion of learning opportunities to maintain or improve GDPs capabilities and confidence in the provision of effective early intervention to prevent dental caries and other oral health problems in infants and toddlers. The general reluctance of GDPs to examine and treat very young children or those with special needs was discussed. According to participants, the quality of undergraduate dental training strongly affects the GDP’s willingness to provide a variety of treatments to children.

CPD courses were also seen as lacking consistency in objectives and content between schools providing CPD. Professional associations also had CPD programs with their own contents. Participants recommended that the provision of CPD courses should also be part of the accreditation of the school. Furthermore, continuing certification is now a mandatory and common practice in many countries. In line with those practices, the group recommended the need for professional registration which includes a mandatory level of CPD points for renewal.

At both level of training (i.e., GDP and specialists), there was consensus that they should be trained to serve as members, leaders and consultants of health/non-health teams. The need to build and maintain working relationships with other discipline workers (e.g., social sciences), to address patients’ needs was emphasised. It was noted that oral conditions, such as ECC and DT, cannot be resolved by one discipline alone, nor by excluding psychological, and social factors. An interdisciplinary approach was seen as essential in addressing the complexity of these conditions and providing and attaining comprehensive health care for the community e community. Integration with teaching and learning disciplines was also highlighted during the discussions. Further, participants noted that, whilst in some dental schools there is a level of integration of PD with other dental disciplines, this was not consistent across the curricula at all schools and faculties.

Disciplines in social sciences (i.e., sociology, anthropology and psychology), nutrition, and *audiolog*y were the most commonly mentioned in which dentists required additional training. The expansion of training on how to communicate with pre-school children and work with their families, other health professionals, local leaders, and the wider community (while applying an evidence-based framework to clinical practice) was also mentioned as an area in need of better coverage. Other health-related disciplines mentioned in which dentists required additional training were kinesiology and public health (e.g. epidemiology, health care management, and health care policy).

Further exposure to research methodology and evidence based practice tools and techniques, and critical appraisal needs to be increased to allow the development of enhanced skills in evidence-based practice. This was seen to be as important with respect to advocacy and leadership.

## Research in paediatric dentistry

Research was recognised as another area of importance and participants agreed on the role of research in providing the scientific basis for PD teaching and practice. Research should be conducted in ways that creates dynamic research environments, networks of collaboration amongst researchers, across disciplines and across the different academic institutions, to maximize resources in ways that can significantly influence health care policies and practice.

Participants uniformly commented on the high quality of researchers who have undertaken PD topics in Chile and in Latin America. Moreover, they highlighted the need to further support, encourage and strengthen the research culture within and amongst Dental Schools to support, attract, train and nurture new cohorts of oral health researchers to develop their capacity for independent research in general and in PD in particular.

When discussing the contents and priorities for a research agenda in PD, the group identified the lack of a national oral health profile that describes the prevalence of oral diseases, risk factors and their distribution in children. Moreover, participants pointed out the need to establish oral health profiles at regional levels (Chile is administratively divided into 15 regions), which would allow for local programming and planning. Scarcity of data was seen by many as a major limitation in their capacity to meet oral health needs [21]. In particular, the need for epidemiological data about ECC and DT was considered central to addressing priorities in oral health set by the Ministry of Health [22], as well as current emphasis on health promotion from the Chilean Government and the Government’s calls for the establishment of baseline oral health data and the strengthening of evaluative efforts [21].

Participants also agreed on the need to develop a resource database to facilitate research, including research funding organizations and opportunities. A database of meeting and research activities was also suggested. The general view was that most oral health research is sponsored and funded by the government and academic centres. For this reason, it was considered important to continue and expand Government and university funding as essential to advancing oral health. In fact, the role of governmental agencies, such as the National Commission for Scientific and Technological Research (*CONICYT*) and the Becas Chile program was seen as fundamental to increasing research and higher (research and professional) degrees in Chile.

However, participants also commented on the lack of a national policy for research and higher degrees supported by dedicated research support teams. The role of the government in establishing national policies was seen as fundamental to developing a strong research culture. In this regard, the example of Brazil was used to illustrate the importance of the role of national policies to reach set objectives in research and higher education.

The role of oral health professional societies (e.g. Society of Paediatric Dentistry, the Dental Association), and the private sector (i.e. industry), with respect to social responsibility and philanthropy were discussed as opportunities for research partnerships. Participants believed that the interactive role between government, professional associations and societies, primary care providers, and universities was synergistic, and a unifying effort to make PD and oral health a priority and to increase resources for oral health care. Participants also noted that private and industry funded research demands continued vigilance regarding research integrity, conflict of interest and academic freedom.

## Opportunities and challenges that paediatric dentistry should not ignore

According to participants, opportunities include the existence of robust knowledge and a body, although limited in number, of well-trained researchers who can provide formal and informal mentoring and role modelling, the availability of national standards and programs, specialist referral centres and specialists with good clinical experience. Even so, a number of challenges are preventing the discipline from achieving its full scientific potential. For example, a general comment was the substantial increase in the number of dental schools and therefore the number of dentists practicing in Chile and a concomitant increase in the number of paediatric dentists, witnessed over the last few years [23]. These workforce issues were of concern, mainly because, according to participants, no modelling or planning had been used with respect to this increase. That was seen as requiring a fundamental and extensive review of general and paediatric dental education, as these programs were characterised by a lack of consistency, integration, interaction and limited resources. As a consequence, clinical experience varies substantially across programs. In most schools, topics in PD emphasised oral pathologies and their treatment, with some focus on ECC. A way forward would be the design of core competencies in both UG and PG education, using coordinated approaches to ensure that contents and curricula are acceptable and adopted.

In addition to limitations in curricula content, there are other structural barriers that need to be reassessed. For example, contractual arrangements and the time assigned to teach do not create the best climate for further development of PD. Part-time staff are effective in teaching, but a high reliance on part-time and casual employment can be less beneficial for the development of a research culture.

Opportunities were also identified in the use of information and communication technologies (ICT). In this regard, participants indicated that there is need to incorporate expansions in education technology to enhance lifelong learning. With the recent explosion of knowledge and technology strategies, the future would undoubtedly involve the use of ICT to supplement both UG and PG, and CPD courses. These tools were mentioned as offering support to personalise the learning experiences through learning design, technology, creative spaces and collaboration.

Standards for education must allow for new types of learning styles and encouragement of distance education/learning, the use of mobile-learning and open content education to achieve quality of education. Distance education, massive open online courses (MOOCs) and web-based learning can facilitate integration and uniform standards across schools and dental faculties. Furthermore, online platforms are essential for program sustainability. Resources can be shared utilising faculties’ time in a cost-effective manner. The simulation learning systems can also integrate technology into the dental course by providing realistic scenarios and supportive learning resources could be used when exposure to real life experiences is not possible.

## Summary

One of the characteristics of the 21^st^ century is the changing makeup of health and non-health professionals involved in meeting the health needs of children and adolescents, and this will continue to evolve [24]. A discussion session was organised to provide information about PD curricula in contemporary education, and as an attempt to generate ideas regarding PD training, education and research. Session participants expressed their concerns about current organization of PD education and the lack of standardisation of training across programs at the different levels of education (i.e., UG, PG or CPD). Early childhood caries and dental trauma represent two common and serious public health problems. To respond to these challenges, dental schools need constant revision, refinement and reformulation to review the validity and reliability of training standards. This forces an ongoing debate on the content of paediatric dentistry curricula to provide direction for improvement of paediatric education at both undergraduate and postgraduate levels. This paper provides suggestions for a way forward for the future of PD in Chile.

The dentist of the future will be expected to treat a range of oral health problems and conditions that affect the individual over their lifespan, with an integrated mix of biomedical and behavioural sciences that need to be an integral part of UG curricula. Dental schools must respond to these needs with innovative evidence-based curricula and enhanced educational methods and experiences that will prepare GDP and PD specialists with the skills and knowledge to deal with these and other new challenges.

The future of PD in Chile, as in any country, must be analysed in the context of a globalised society, with constant ethical, technological, epidemiologic, educational and sociological changes and challenges [21]. The need to understand these aspects forces a debate, from time to time, on the content of PD curricula. Thus, the PD curriculum requires constant revision, refinement and reformulation to establish the validity and reliability of training standards.

The workshop’s objective was to generate discussion and debate among the dental schools represented in this session. It is hoped that the results of these discussions provide direction for improvements of paediatric dentistry education at both undergraduate and postgraduate levels, and also a way forward for the future of PD in Chile. The accreditation process, regulated by the National Accreditation Commission (CNA), considers several aspects that programs must demonstrate and the work and inputs from the various programs represented in this session provided an initial step to contribute to this process. More research and discussion are necessary to document and assess adequacy of PD programs. Many countries are facing similar public health challenges. Thus, it was considered appropriate to present these results for further research and planning. Session organisers invite debate, support, and challenging of the ideas expressed, and hope that the discussions from the meeting serve as a forum to develop a consensus for educational innovation and development of a model curriculum in Paediatric Dentistry. Additionally, a proposal will be submitted for the next Congress of the International Association of Paediatric Dentistry, for a dedicated session to further discuss paediatric education and accreditation of programs in Chile.

## Abbreviations

CNA, National Accreditation Commission; CONICYT, Commission for Scientific and Technological Research; CPD, continuing professional development; DT, dental trauma; ECC, early childhood caries; GDP, general dental practitioner; ICT, information and communication technologies; MOOC, massive open online courses; PD, paediatric dentistry; PG, post-graduate/graduate; UG, undergraduate

## Footnotes

1 In Chile, there is a national accreditation board. Nonetheless, no standard curriculum in Chilean dental schools exists. For UG training, the accreditation board requires a concordance between curriculum contents and source objectives, but no specific core contents. In 2014, after this meeting, the accreditation for dental specialties was implemented, but only one is accredited for PG paediatric dentistry training.
